# Preoperative Anxiety Is Associated with Early Postoperative Pain After Arthroscopic Ankle Ligament Reconstruction: A Prospective Cohort Study

**DOI:** 10.3390/jcm15124656

**Published:** 2026-06-16

**Authors:** Ryan Bou Raad, Mohamad Moussa, Gregoire Rougereau, Eugenie Valentin, Olivier Grimaud, Frederic Khiami, Nicolas Lefevre, Alexandre Hardy

**Affiliations:** 1 Centre Hospitalier Jeanne de Navarre, 02400 Château-Thierry, France; 2 Groupe Hospitalier Selestat-Obernai, 67600 Selestat, France; mhamadmoussa71976798@gmail.com; 3 Ramsay Santé, Clinique du Sport, 75005 Paris, France; greg.rougereau@gmail.com (G.R.); eugenie@chirurgiedusport.com (E.V.); olivgrimaud@hotmail.com (O.G.); frederic.khiami@ahparis.org (F.K.); dr.lefevre@chirurgiedusport.com (N.L.); alexandre.hardy@me.com (A.H.)

**Keywords:** preoperative anxiety, arthroscopic ankle ligament reconstruction, visual analog scale, surgical fear questionnaire, patient acceptable symptom state, postoperative pain

## Abstract

**Purpose**: We evaluate the relationship between preoperative anxiety, as measured by the Surgical Fear Questionnaire (SFQ), and postoperative pain intensity during the first three days following arthroscopic ankle ligament reconstruction. A secondary objective was to determine whether preoperative anxiety levels are associated with failure to reach the Patient Acceptable Symptom State (PASS) threshold for pain by postoperative day 3. **Methods**: A prospective cohort of 68 patients undergoing anatomic arthroscopic ankle lateral ligament reconstruction between January 2023 and March 2025 was assessed. Patients were categorized into high- and low-anxiety groups based on a tertile distribution of SFQ scores, with a threshold of 45. Pain intensity was measured using the Visual Analog Scale (VAS) on days 0, 1, 2, and 3 postoperatively. The proportion of patients with unacceptable pain (VAS > 3) according to the patient’s acceptable symptom state was compared between groups. A receiver operating characteristic (ROC) curve identified the optimal SFQ threshold for discriminating PASS achievement on day 3. Odds ratios were calculated using logistic regression. **Results**: A total of 68 patients were included (34 in each group). The mean age was 32.9 ± 10.5 years in the high-anxiety group and 33.1 ± 9.9 years in the low-anxiety group (*p* = 0.747). The mean SFQ score was 58.9 ± 11.9 in the high-anxiety group and 28.0 ± 10.8 in the low-anxiety group (*p* < 0.001). There were no significant differences between groups in demographic characteristics. Patients in the high-anxiety group reported significantly higher VAS scores at all postoperative time points. The proportion failing to achieve PASS on day 3 was also significantly greater in the high-anxiety group (67.6% vs. 35.3%, *p* = 0.015). An SFQ threshold of 46 discriminated PASS status with an AUC of 0.70. Patients with SFQ ≥ 46 had more than triple the odds of not achieving PASS (OR = 3.39; 95% CI: 1.26–9.59, *p* = 0.017). **Conclusions**: Preoperative anxiety is significantly associated with early postoperative pain and pain acceptability following arthroscopic ankle ligament reconstruction. Identifying and managing high-anxiety patients preoperatively may improve pain outcomes and patient satisfaction.

## 1. Introduction

Postoperative pain is a significant concern following arthroscopic ankle ligament reconstruction for ankle instability, with recovery outcomes varying greatly among patients [1,2,3,4]. While physical factors such as tissue healing and surgical techniques [5] are essential in recovery [6], psychological factors like preoperative anxiety or psychological readiness to return to sport [7] can also play a crucial role in how patients experience pain and recover post-surgery.

Anxiety is a factor that has been consistently associated with increased pain perception and delayed recovery in various surgical settings [8,9,10]. Preoperative anxiety refers to the emotional and psychological distress experienced by patients before undergoing surgery. This anxiety can manifest as fear of the unknown, worry about potential complications, or concern about post-surgical recovery, including pain, mobility, and the impact on daily life [6].

Preoperative anxiety is common and can affect how patients cope with pain after surgery, potentially leading to greater postoperative pain and a prolonged recovery process [11]. The heightened emotional state and increased focus on pain during recovery can exacerbate the experience of discomfort and increase pain sensitivity, leading to an overestimation of pain severity and slower rehabilitation [8,9]. Furthermore, anxiety can interfere with the patient’s ability to engage in rehabilitation activities, which are crucial for recovery [12].

While postoperative pain after foot and ankle surgery has been described in previous studies [1], and psychological factors such as anxiety have been associated with pain perception in orthopedic populations [8,9,10], data specifically addressing the relationship between preoperative anxiety and early postoperative pain during the first days following arthroscopic ankle ligament reconstruction remain limited. This is particularly relevant in a young and active population in whom early postoperative pain may influence mobilization and return to activity.

This study aims to investigate the role of preoperative anxiety, measured by the Surgical Fear Questionnaire (SFQ) [13,14], in its association with postoperative pain levels following arthroscopic ankle ligamentoplasty. The primary objective is to examine the correlation between preoperative anxiety and postoperative pain, measured by the Visual Analog Scale (VAS) at multiple time points over the first three days following surgery (D0, D1, D2, D3).

As a secondary objective, the study will explore whether preoperative anxiety correlates with whether patients find their postoperative pain acceptable using the PASS score based on their VAS ratings.

We hypothesized that patients with higher preoperative anxiety, as measured by the Surgical Fear Questionnaire (SFQ), would experience higher levels of postoperative pain and a lower likelihood of achieving the Patient Acceptable Symptom State (PASS) during the early recovery period following anatomic arthroscopic ankle lateral ligament reconstruction.

## 2. Materials and Methods

### 2.1. Patients

This study was designed as a prospective cohort study. A propensity score-matched subgroup analysis was pre-specified to compare patients with high versus low preoperative anxiety.

This prospective study included patients who underwent anatomic arthroscopic ankle lateral ligament reconstruction at a specialized sports surgery referral center between January 2023 and March 2025.

### 2.2. Inclusion and Exclusion Criteria

Patients were eligible for inclusion if they were over 18 years of age, presented with chronic lateral ankle instability, had a positive talar tilt test on clinical examination, and MRI-confirmed ligamentous lesions. All patients had failed a course of conservative treatment prior to surgical intervention. Patients were excluded if they underwent revision surgery, refused to provide informed consent, were lost to follow-up during the postoperative assessment period, or had incomplete preoperative or postoperative data, including the SFQ or VAS. Patients receiving antidepressant treatment at the time of surgery were also excluded. No formal a priori sample size calculation was performed, as all consecutive eligible patients during the study period were included.

The study was approved by the Institutional Review Board, and informed consent was obtained from all participants prior to their inclusion in the study.

### 2.3. Surgical Technique

All patients underwent arthroscopic anatomical reconstruction of the anterior talofibular and calcaneofibular (CFL) ligaments using the technique described by Lopes et al. [5]. All patients received spinal anesthesia combined with a sciatic nerve block. This procedure involved gracilis tendon harvesting through a short medial approach. The harvested graft was soaked in a vancomycin solution before implantation [15]. Standard anteromedial and anterolateral arthroscopic portals were used for joint inspection and tunnel preparation. The graft was fixed to the talus using a Bio-Tenodesis screw and to the fibula using a cortical endobutton. A full-thickness calcaneal tunnel was created percutaneously to restore the CFL insertion. Final graft tension was adjusted with the ankle in neutral position, and fixation to the calcaneus was performed under arthroscopic guidance using a second Bio-Tenodesis screw (Arthrex, Naples, FL, USA).

### 2.4. Postoperative Pain Management Protocol

Patients were allowed immediate weightbearing in a walking boot, worn continuously for the first three weeks. Cryotherapy was prescribed three times daily to manage swelling. The analgesic regimen included paracetamol (500 mg, four times per day) and Lamaline (paracetamol 300 mg, caffeine 30 mg, opium 10 mg) up to four times daily, depending on pain severity. In case of inadequate pain control, oral nefopam (30 mg, up to every 6 h) was prescribed as a complementary analgesic. Omeprazole (20 mg daily) was co-prescribed as gastric protection during the 5-day course of oral celecoxib (200 mg twice daily). Doses were adapted to body weight as indicated in the medical prescription. This standardized multimodal analgesic protocol was applied to all patients, with dose adjustments based on individual tolerance and clinical need.

### 2.5. Preoperative Surgical Fear Questionnaire (SFQ)

Preoperative anxiety levels were assessed using the Surgical Fear Questionnaire (SFQ), a validated tool designed to measure anxiety related to surgery [14]. The SFQ consists of eight items that assess patients’ fear of short-term and long-term surgical consequences. Scores range from 0 to 64, with higher scores indicating greater levels of anxiety. The SFQ was administered during the preoperative assessment.

### 2.6. Outcome Measures

The primary outcomes of the study were postoperative pain intensity, measured using the Visual Analog Scale (VAS), and pain acceptability, evaluated using the Patient Acceptable Symptom State (PASS).

### 2.7. Visual Analog Scale (VAS)

Postoperative pain intensity was measured using the Visual Analog Scale (VAS), a widely used method for quantifying pain [3,16,17,18]. The VAS is a 100 mm line, where one end represents no pain and the other end represents the worst pain imaginable. Patients marked the line according to their perceived pain intensity, and the distance from the “no pain” end to the patient’s mark was measured in millimeters to quantify pain intensity. VAS scores were recorded at five time points: immediately post-surgery (day 0), during the night of the surgery day (night 0), and subsequently each morning of postoperative day 1 (day 1), day 2 (day 2), and day 3 (day 3).

### 2.8. The Patient Acceptable Symptom State (PASS)

The Patient Acceptable Symptom State (PASS) was used to determine whether postoperative pain levels were considered acceptable from the patient’s perspective. In this study, the threshold for acceptable pain was set at a Visual Analog Scale (VAS) score of ≤3, consistent with established benchmarks in the literature [12,19,20]. This binary outcome was used to classify patients as either having achieved or not achieved an acceptable symptom state at each postoperative time point.

Secondary outcomes included the occurrence of perioperative side effects and the progression of patient mobilization. Side effects were recorded daily from day 0 to day 3 and included nausea, dizziness, malaise, and acute anxiety symptoms, based on patient self-reports and nursing assessments.

Mobilization was defined as the patient’s ability to get up and ambulate, with or without assistance. It was recorded as a binary outcome at each time point (day 0 to day 3) and reflected the patient’s functional recovery trajectory during the early postoperative period.

### 2.9. Data Collection

All patients were evaluated preoperatively, and postoperative follow-up assessments were conducted in the immediate postoperative phase (D0) and at postoperative days 1, 2, and 3 (D1, D2, and D3).

All patient-reported outcome measures (PROMs) were completed directly by the patients using the secure digital platform WebSurvey. Patients filled out the questionnaires independently, without any influence or guidance from clinical staff or data collectors. Preoperative data included age, sex, body mass index (BMI), activity level, surgical laterality, CAIT score, and baseline PROMs including the SFQ, Foot and Ankle Outcome Score (FAOS), Foot and Ankle Ability Measure (FAAM), and Ankle Ligament Reconstruction–Return to Sport after Injury scale (ALR-RSI). Postoperative data were collected at four time points (D0 to D3) and included VAS, presence of side effects, and daily mobilization status.

### 2.10. Participants and Flow Chart

**Figure 1:**
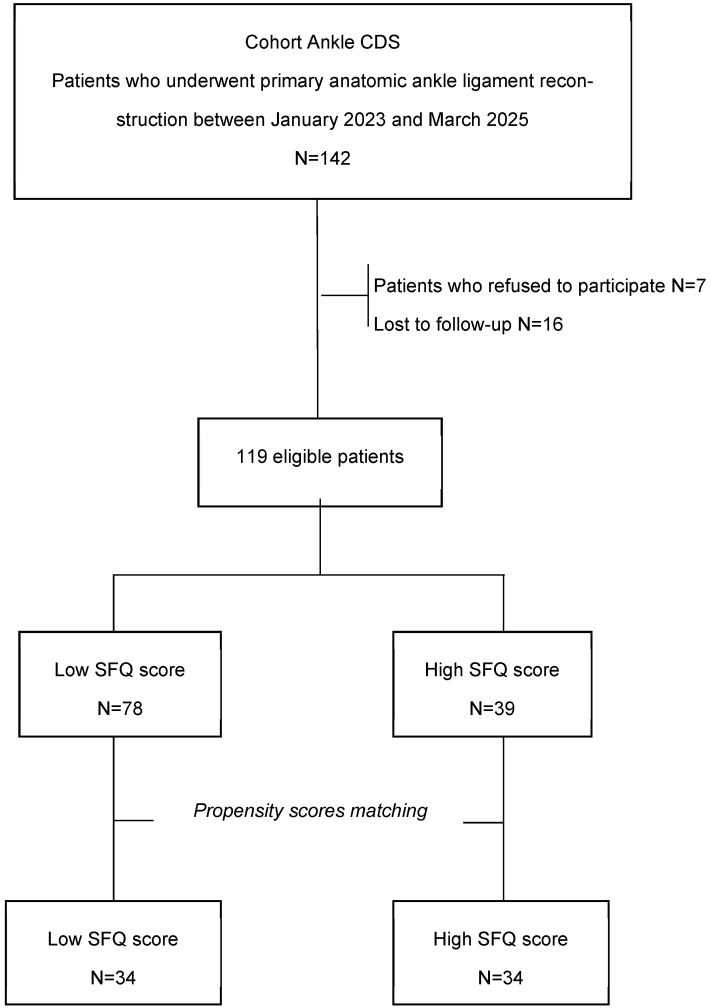
Flow chart.

During the study timeframe, 142 patients underwent arthroscopic ankle ligament reconstruction. Of these, 23 patients were excluded: 7 declined to participate and 16 were lost to follow-up. A total of 119 patients were eligible for analysis. To categorize patients into anxiety-based groups, the tertile distribution of the Surgical Fear Questionnaire (SFQ) score was used. The top 33% of patients (SFQ > 45) were classified as the high-anxiety group. The remaining 66% of patients comprised the low-anxiety group. This grouping was based on tertile distribution and is distinct from the ROC-derived threshold used for predictive analysis. A 1:1 matching procedure was then applied to form two comparable groups of 34 patients each. The flow chart of patient inclusion and allocation is presented in Figure 1.

### 2.11. Statistical Analysis

A propensity score matching procedure was performed between the high and low-anxiety groups based on age, sex, and preoperative Cumberland Ankle Instability Tool (CAIT) score. Matching was conducted to ensure comparability between groups and to reduce potential confounding factors that could affect the association between preoperative anxiety and postoperative outcomes.

Age, sex, and preoperative CAIT score were selected a priori as clinically relevant variables reflecting baseline patient characteristics and ankle instability severity. Other preoperative PROMs were not included in the model to avoid overfitting given the sample size.

The propensity score was derived using a logistic regression model, and participants were matched at a 1:1 ratio with a caliper width of 0.2 to minimize selection bias.

Descriptive statistics were calculated for each group, including means and standard deviations (SD) for continuous variables, or median and interquartile range (IQR) when appropriate, and frequencies and percentages for categorical variables. Normality of continuous variables was assessed prior to analysis, and appropriate parametric or non-parametric tests were applied accordingly.

Comparative statistical analyses were performed to identify significant differences between the two groups. For continuous variables, independent *t*-tests were used if the data were normally distributed, while the Mann–Whitney U test was applied for non-normally distributed data. For categorical variables, comparisons were made using the Chi-square test or Fisher’s exact test when appropriate. Following matching, comparisons between groups were performed using unpaired statistical tests.

To identify the optimal SFQ threshold for discriminating PASS status at 3 days postoperation, a ROC curve analysis was performed. An area under the curve (AUC) of 0.5 indicates no discriminative ability, 0.7–0.8 is acceptable, 0.8–0.9 is good, and above 0.9 is excellent [21]. The optimal threshold was determined using the Youden Index, which maximizes sensitivity and specificity. This approach allowed us to empirically derive the stratification threshold based on our dataset. Finally, odds ratios (ORs) with 95% confidence intervals (CI) were calculated using logistic regression to assess the association between the threshold groups and the probability of passing the PASS at postoperative day 3.

Logistic regression analysis was performed as an unadjusted model, as baseline characteristics were balanced through the matching procedure. A *p*-value of <0.05 was considered statistically significant for all comparisons. Repeated postoperative pain measurements were analyzed using a linear mixed-effects model including SFQ group, postoperative time point, and their interaction as fixed effects, with patient identifier included as a random effect to account for within-subject correlations.

## 3. Results

A total of 68 patients were included in the analysis, with 34 patients in each group (high anxiety vs. low anxiety) after matching by age, sex, and preoperative CAIT score. No missing data were observed for postoperative VAS scores or PASS outcomes at any postoperative time point. There were no significant differences between the two groups in demographic characteristics, including BMI, activity level, or laterality of the procedure (Table 1).

VAS scores were consistently higher in the high-anxiety group across all postoperative days, except for the first postoperative night (Table 2). On day 0, the high-anxiety group reported a mean VAS score of 6.1 ± 3.0 compared to 4.5 ± 3.1 in the low-anxiety group (*p* = 0.036). On day 1, the scores were 5.6 ± 2.7 and 4.2 ± 2.5, respectively (*p* = 0.030). This difference persisted on day 2 (5.0 ± 2.6 vs. 3.6 ± 2.6, *p* = 0.037) and day 3 (4.5 ± 2.6 vs. 3.1 ± 2.5, *p* = 0.033). Linear mixed-effects analysis demonstrated a significant overall effect of SFQ group on postoperative pain scores (*p* = 0.024), as well as a significant effect of time (*p* < 0.001). However, the interaction between SFQ group and time was not significant (*p* = 0.98), suggesting a similar postoperative pain trajectory over time in both groups.

The proportion of patients exceeding the PASS threshold (VAS > 3) was significantly higher in the high-anxiety group on multiple postoperative days (Table 3). On day 0, 44.1% of high-anxiety patients did not achieve PASS, compared to 17.6% of the low-anxiety group (*p* = 0.034). This trend was also observed on day 2 (61.8% vs. 29.4%, *p* = 0.014) and day 3 (67.6% vs. 35.3%, *p* = 0.015), while the difference on day 1 was not statistically significant.

Perioperative side effects and mobilization data are summarized in Table 4 and should be interpreted as exploratory findings. On day 0, significantly fewer side effects were reported in the high-anxiety group compared to the low-anxiety group (88.2% vs. 52.9% reported no side effects, *p* = 0.008). By day 3, all patients in the high-anxiety group had mobilized (100%) compared to 72.3% in the low-anxiety group (*p* = 0.005). Additionally, fewer patients in the high-anxiety group reported side effects on day 3 (79.4% vs. 55.9%, *p* = 0.048).

**Figure 2:**
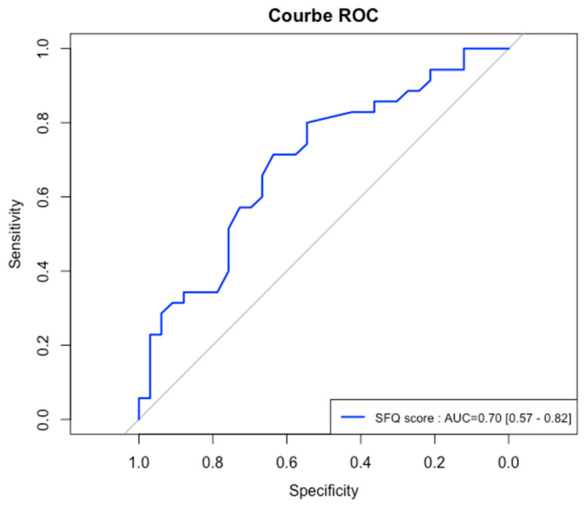
ROC curve.

Receiver operating characteristic (ROC) (Figure 2) curve analysis identified a preoperative SFQ cutoff value of 46 as optimal for discriminating PASS status at day 3 using the Youden index (Se = 71.4%, Sp = 63.6%). The area under the curve (AUC) was 0.70 (95% CI [0.57–0.82]), indicating acceptable discriminative ability (Table 5).

Patients with an SFQ score ≥ 46 had significantly higher odds of failing to reach the PASS threshold by day 3 (OR = 3.39; 95% CI: 1.26–9.59; *p* = 0.017).

## 4. Discussion

This study demonstrates that preoperative anxiety, as measured by the Surgical Fear Questionnaire (SFQ), is significantly associated with postoperative pain perception following arthroscopic ankle ligament reconstruction. Patients with higher levels of anxiety prior to surgery experienced significantly greater pain levels in the immediate postoperative period, continuing through the first three days post-surgery. This observation aligns with previous studies showing that psychological factors, particularly anxiety, are strongly correlated with pain perception and recovery outcomes in surgical populations [8,10].

Patient Reported Outcomes Measurement Information System (PROMIS) scores have been shown to correlate with PASS thresholds in foot and ankle surgery, validating PASS as a clinically meaningful marker [22]. Our data also show that this association was already evident on day 0 and day 2, not only on day 3 following ankle arthroscopy. The cutoff of 46 was not informed by the prior literature but emerged from our ROC analysis as the most discriminative value for PASS achievement on day 3. The cutoff of 46, derived from ROC analysis, should be considered exploratory and data-driven and requires external validation before being used in clinical practice.

This highlights the early influence of anxiety on pain acceptability immediately after surgery.

Higher patient expectations have also been associated with increased postoperative pain scores [23], reinforcing the need to understand how psychological factors shape recovery experiences.

Interestingly, patients in the high-anxiety group reported fewer side effects on day 0 and were more likely to mobilize by day 3. These findings should be interpreted cautiously, as they were exploratory and not primary outcomes. This unexpected finding contrasts with prior work by Ahn and Cho [24], who identified preoperative anxiety and other psychological factors as contributors to persistent postoperative pain after technically successful ankle surgery. While both studies highlight the role of anxiety in shaping recovery, our findings suggest that anxiety may also drive early mobilization and altered symptom reporting—potentially reflecting a short-term behavioral adaptation rather than long-term functional improvement. Indeed, this earlier mobilization in anxious patients may reflect heightened vigilance or a compensatory coping mechanism, wherein patients attempt to proactively manage their postoperative concerns through increased activity. Rather than indicating a true functional benefit, this behavior could represent an adaptive yet potentially maladaptive response to anxiety. Future research incorporating qualitative methodologies might better elucidate the psychological motivations behind such early mobilization behaviors.

Although the difference in pain scores on the first postoperative night (night 0) did not reach statistical significance, the trend was consistent with subsequent days, with higher pain levels reported in the high-anxiety group.

The findings of this study align with the broader literature on anxiety and pain catastrophizing in orthopedic surgery. Papaioannou et al. [25] and Gibson et al. [9] emphasize the role of catastrophizing in exacerbating pain perception, and while we did not directly measure catastrophizing in our study, anxiety itself is a well-documented precursor to such cognitive distortions. Our results suggest that addressing preoperative anxiety may improve postoperative outcomes, as suggested by Baumhauer et al. [26], who observed better recovery when anxiety levels were managed before surgery.

The odds ratio analysis provides further evidence that preoperative anxiety is an associated factor for postoperative pain management, as patients in the high-anxiety group had significantly higher odds of failing to achieve the PASS score. This finding has important clinical implications, as it suggests that interventions targeting preoperative anxiety may be beneficial in improving recovery outcomes and patient satisfaction after surgery, as suggested in the literature [27]. Evidence-based strategies such as brief cognitive behavioral therapy (CBT), structured preoperative education sessions, and mobile-based anxiety management tools have been shown to reduce surgical fear and may help mitigate early postoperative pain in high-anxiety patients [28,29,30,31]. From a practical standpoint, identifying high-anxiety patients preoperatively may help tailor perioperative management, including reinforced patient education and early rehabilitation strategies.

Our study has several limitations. First, the follow-up period was limited to the first three postoperative days, preventing assessment of longer-term outcomes. Second, although we categorized patients based on their preoperative SFQ scores, other psychological factors such as pain catastrophizing or self-efficacy were not evaluated. Finally, the sample size was modest after propensity score matching, which may limit statistical power. Further research is needed to determine how psychological interventions aimed at reducing preoperative anxiety may influence both pain outcomes and rehabilitation adherence.

## 5. Conclusions

In conclusion, this study suggests that preoperative anxiety is associated with increased postoperative pain perception following arthroscopic ankle ligament reconstruction. Identifying and addressing high-anxiety levels before surgery may represent an important target for personalized pain management. Psychological screening and early interventions could contribute to improved patient satisfaction, reduced pain, and better adherence to rehabilitation in the early postoperative period.

## Figures and Tables

**Table 1:** Description of the study population.

	Overall	High-Anxiety Group	Low-Anxiety Group	*p*-Value
	N = 68	N = 34	N = 34	
Age at surgery	33.0 (10.1)	32.9 (10.5)	33.1 (9.9)	0.747
Sex				>0.999
Female	44 (64.7%)	22 (64.7%)	22 (64.7%)	
Male	24 (35.3%)	12 (35.3%)	12 (35.3%)	
BMI	25.1 (5.5)	25.5 (6.1)	24.8 (4.8)	0.624
Operated ankle side				0.222
Right	38 (55.9%)	16 (47.1%)	22 (64.7%)	
Left	30 (44.1%)	18 (52.9%)	12 (35.3%)	
Preoperative SFQ Score	43.4 (19.2)	58.9 (11.9)	28.0 (10.8)	<0.001
Professional activity level				0.748
Physical	28 (41.2%)	13 (38.2%)	15 (44.1%)	
Sedentary	17 (25.0%)	10 (29.4%)	7 (20.6%)	
Semi-sedentary	23 (33.8%)	11 (32.4%)	12 (35.3%)	
Level of sport				0.158
Competition	18 (26.5%)	6 (17.6%)	12 (35.3%)	
Occasional leisure	15 (22.1%)	9 (26.5%)	6 (17.6%)	
Regular leisure	27 (39.7%)	16 (47.1%)	11 (32.4%)	
Professional	5 (7.4%)	3 (8.8%)	2 (5.9%)	
Sedentary	3 (4.4%)	0 (0.0%)	3 (8.8%)	
Type of sport				0.051
No sport	7 (10.3%)	3 (8.8%)	4 (11.8%)	
Linear sport (jogging, cycling, etc.)	12 (17.6%)	10 (29.4%)	2 (5.9%)	
Pivot sport (ski, tennis, squash, badminton, volleyball, dance, gymnasium, golf)	26 (38.2%)	13 (38.2%)	13 (38.2%)	
Contact pivot sport (Football, rugby, basketball, handball, hockey, etc.)	23 (33.8%)	8 (23.5%)	15 (44.1%)	
Preoperative FAOSs				
Pain score	52.0 (26.8)	50.3 (27.2)	53.8 (26.7)	0.633
Stiffness score	54.9 (19.5)	52.5 (17.8)	57.1 (21.1)	0.255
Quality of life score	29.5 (16.1)	28.0 (17.8)	30.9 (14.3)	0.350
Preoperative FAAM scores	63.4 (18.0)	59.8 (18.3)	67.0 (17.3)	0.085
Activity score	72.2 (18.3)	68.3 (19.1)	76.1 (17.0)	0.068
Sport score	39.3 (22.1)	36.4 (22.1)	42.1 (22.0)	0.303
Pre-operative CAIT score	8.6 (4.8)	7.6 (4.4)	9.6 (5.1)	0.099
Stage				0.472
Stage 0: Normal and continuous ATFL	2 (2.9%)	1 (2.9%)	1 (2.9%)	
Stage 2: Detachment of the ATFL	17 (25.0%)	8 (23.5%)	9 (26.5%)	
Stage 3: Thinned ligament, with or without scar tissue	23 (33.8%)	14 (41.2%)	9 (26.5%)	
Stage 4: Scar tissue without residual ligament	26 (38.2%)	11 (32.4%)	15 (44.1%)	
Grade categories				0.614
Grades 0 or 1	64 (94.1%)	31 (91.2%)	33 (97.1%)	
Grades 2, 3 or 4	4 (5.9%)	3 (8.8%)	1 (2.9%)	

**Table 2:** Comparison of pain between the two groups.

	High SFQ Group	Low SFQ Group	Mean Difference	CI (95%)	*p*-Value
VAS score day 0, mean (sd) median (Q1–Q3)	6.1 (3.0) 6.0 (4.0–8.0)	4.5 (3.1) 4.0 (2.0–7.0)	1.6	[0.3; 2.9]	0.036
VAS score night 0, mean (sd) median (Q1–Q3)	5.8 (2.9) 6.0 (4.0–8.0)	4.4 (3.0) 5.0 (2.0–7.0)	1.4	[0.1; 2.7]	0.069
VAS score day 1, mean (sd) median (Q1–Q3)	5.6 (2.7) 6.0 (4.0–7.0)	4.2 (2.5) 5.0 (3.0–6.0)	1.4	[0.2; 2.6]	0.030
VAS score day 2, mean (sd) median (Q1–Q3)	5.0 (2.6) 6.0 (3.0–7.0)	3.6 (2.6) 3.0 (2.0–6.0)	1.4	[0.2; 2.6]	0.037
VAS score day 3, mean (sd) median (Q1–Q3)	4.5 (2.6) 5.0 (2.0–6.0)	3.1 (2.5) 3.0 (1.0–4.0)	1.4	[0.2; 2.6]	0.033

**Table 3:** Percentage of patients crossing the PASS threshold for VAS.

	Low SFQ Group	High SFQ Group	*p*-Value
Day 0	6 (17.6%)	15 (44.1%)	0.034
First night	7 (20.6%)	14 (41.2%)	0.114
Day 1	8 (23.5%)	14 (41.2%)	0.194
Day 2	10 (29.4%)	21 (61.8%)	0.014
Day 3	12 (35.3%)	23 (67.6%)	0.015

**Table 4:** Side effects.

	Low SFQ Group	High SFQ Group	*p*-Value
Day 0			
Did you get up?			0.294
Yes	21 (61.8%)	26 (76.5%)	
No	13 (38.2%)	8 (23.5%)	
Side effects			0.008
None	18 (52.9%)	30 (88.2%)	
Anxiety	3 (8.8%)	0 (0.0%)	
Other	3 (8.8%)	0 (0.0%)	
Malaise	2 (5.9%)	0 (0.0%)	
Nausea and vomiting	5 (14.7%)	1 (2.9%)	
Dizziness	3 (8.8%)	3 (8.8%)	
Night 0			
Side effects			0.087
None	19 (55.9%)	29 (85.3%)	
Anxiety	3 (8.8%)	1 (2.9%)	
Other	4 (11.8%)	2 (5.9%)	
Malaise	1 (2.9%)	0 (0.0%)	
Nausea and vomiting	6 (17.6%)	1 (2.9%)	
Dizziness	1 (2.9%)	1 (2.9%)	
Day 1			
Did you get up?			0.362
Yes	28 (72.3%)	32 (94.1%)	
No	6 (17.6%)	2 (5.9%)	
Side effects			0.057
None	14 (41.2%)	23 (67.6%)	
Anxiety	3 (8.8%)	0 (0.0%)	
Other	3 (8.8%)	4 (11.8%)	
Malaise	2 (5.9%)	1 (2.9%)	
Nausea and vomiting	7 (20.6%)	1 (2.9%)	
Dizziness	5 (14.7%)	5 (14.7%)	
Day 2			
Did you get up?			0.259
Yes	29 (85.3%)	33 (94.1%)	
No	5 (14.7%)	2 (5.9%)	
Side effects			0.172
None	17 (50.0%)	25 (73.5%)	
Anxiety	0 (0.0%)	1 (2.9%)	
Other	7 (20.6%)	3 (8.8%)	
Stomach pain	0 (0.0%)	1 (2.9%)	
Malaise	1 (2.9%)	0 (0.0%)	
Nausea and vomiting	5 (14.7%)	2 (5.9%)	
Dizziness	4 (11.8%)	2 (5.9%)	
Day 3			
Did you get up?			0.005
Yes	28 (72.3%)	34 (100.0%)	
No	6 (17.6%)	0 (0.0%)	
Side effects			0.048
None	20 (58.8%)	29 (85.3%)	
Anxiety	1 (2.9%)	0 (0.0%)	
Other	4 (11.8%)	3 (8.8%)	
Stomach pain	0 (0.0%)	1 (2.9%)	
Malaise	1 (2.9%)	0 (0.0%)	
Nausea and vomiting	4 (11.8%)	0 (0.0%)	
Dizziness	4 (11.8%)	1 (2.9%)	

**Table 5:** ROC curve analysis.

	OR	95% CI	*p*-Value
Group (Threshold ≥ 46)	3.39	1.26, 9.59	0.017

## Data Availability

Data is unavailable due to privacy or ethical restrictions.

## Abbreviations

The following abbreviations are used in this manuscript:

VAS	Visual analog scale
SFQ	Surgical Fear Questionnaire
ROC	Receiver operating characteristic
PASS	Patient Acceptable Symptom State
